# An Ad/MVA vectored *Theileria parva* antigen induces schizont-specific CD8^+^ central memory T cells and confers partial protection against a lethal challenge

**DOI:** 10.1038/s41541-018-0073-5

**Published:** 2018-09-11

**Authors:** Nicholas Svitek, Rosemary Saya, Elias Awino, Stephen Munyao, Robert Muriuki, Thomas Njoroge, Roger Pellé, Nicholas Ndiwa, Jane Poole, Sarah Gilbert, Vishvanath Nene, Lucilla Steinaa

**Affiliations:** 1 International Livestock Research Institute (ILRI), P.O. Box 30709, 00100 Nairobi, Kenya; 2 The Jenner Institute, University of Oxford, Old Road Campus Research Building, Roosevelt Drive Oxford, OX3 7DQ UK

## Abstract

The parasite *Theileria parva* is the causative agent of East Coast fever (ECF), one of the most serious cattle diseases in sub-Saharan Africa, and directly impacts smallholder farmers’ livelihoods. There is an efficient live-parasite vaccine, but issues with transmission of vaccine strains, need of a cold chain, and antibiotics limit its utilization. This has fostered research towards subunit vaccination. Cytotoxic T lymphocytes (CTL) are crucial in combating the infection by lysing *T. parva*-infected cells. Tp1 is an immunodominant CTL antigen, which induces Tp1-specific responses in 70–80% of cattle of the A18 or A18v haplotype during vaccination with the live vaccine. In this study, human adenovirus serotype 5 (HAd5) and modified vaccinia Ankara (MVA) were assessed for their ability to induce Tp1-specific immunity. Both viral vectors expressing the Tp1 antigen were inoculated in cattle by a heterologous prime-boost vaccination regimen. All 15 animals responded to Tp1 as determined by ELISpot. Of these, 14 reacted to the known Tp1 epitope, assayed by ELISpot and tetramer analyses, with CTL peaking 1-week post-MVA boost. Eleven animals developed CTL with specific cytotoxic activity towards peripheral blood mononuclear cells (PBMC) pulsed with the Tp1 epitope. Moreover, 36% of the animals with a Tp1 epitope-specific response survived a lethal challenge with *T. parva* 5 weeks post-MVA boost. Reduction of the parasitemia correlated with increased percentages of central memory lymphocytes in the Tp1 epitope-specific CD8^+^ populations. These results indicate that Tp1 is a promising antigen to include in a subunit vaccine and central memory cells are crucial for clearing the parasite.

## East Coast fever: Developing an accessible vaccine for African farmers

A vaccine expressing parasitic proteins offers more convenient East Coast fever prophylaxis. Current vaccination for the cattle disease, caused by the parasite *Theileria parva* and a detriment to sub-Saharan African farmers, involves inconvenient injection with live parasites before antibiotic treatment (ITM). A collaboration led by Nicholas Svitek, of the Kenyan International Livestock Research Institute, designed a candidate to provoke cellular immune responses against the parasitic antigen Tp1—an ITM vaccine candidate. In tests on cattle, 93% created Tp1-targeting T cells, and 33% survived a lethal dose of *T. parva*. The East Coast fever reduction seen in animals in this research outperformed a recent study and was able to generate the same immune memory cells that ITM inspires to provide long-lasting protection. Future research might integrate more antigens with this Tp1 vaccine to provide more comprehensive protection.

## Introduction

East Coast fever (ECF) is one of the most devastating tick-borne infectious diseases of cattle in sub-Saharan Africa. ECF has been targeted, by the Food and Agriculture Organization of the United Nations (FAO) and by the World Organization for Animal Health (OIE), as a high priority infectious disease for control to improve livelihoods of poor smallholder farmers.^1^ The burden associated with this disease is heavy, with mortalities reaching 70% in susceptible populations of eastern, central, and southern Africa where ECF is endemic.^2^ Even if animals survive the infection, milk and meat production is greatly affected^3,4^ and ECF has therefore a substantial socio-economic impact. Hence, a vaccine to control this disease is of high value in affected countries and will certainly lead to improved human lives.^5^

This lymphoproliferative disease is caused by a tick-borne protozoan parasite, *Theileria parva*, which infects African buffaloes and cattle, but only the latter develop disease. There is a commercially available live vaccine, which is based on injection of a cocktail of three sporozoites isolates, called the Muguga cocktail, followed by treatment with long-acting tetracycline,^6–8^ known as the “Infection-and-Treatment Method” (ITM). The ITM acts similar to a live-attenuated vaccine (LAV), and it has therefore the advantage of providing long-lived immunity^9,10^ as observed with other LAV such as the yellow fever, measles, or Rinderpest vaccines.^11,12^ However, several drawbacks limit broad utilization of this vaccine. For example, it possesses the risk of spreading the disease to naïve populations as vaccinated animals can become carriers of the parasite.^13^ This has particularly impeded a broad deployment of this vaccine in several African countries due to differences in circulating strains. Other obstacles that hinder a broad utilization include the high costs associated with the use of antibiotics included in the ITM procedure, the need for a liquid nitrogen cold chain to deliver the vaccine, and the complexity and regulatory aspects associated with its production. Therefore, there is an urgent need for a more affordable, simpler, and safer vaccine.

Substantial evidence indicates that cytotoxic CD8^+^ T lymphocytes are crucial for protection of animals towards this parasite.^9,14–16^ It has been shown that the schizont stage, which is the pathogenic stage of the parasite, is the target of cytotoxic T lymphocytes (CTL) elicited by the ITM vaccination.^17–19^ In addition, early studies showed that adoptive transfer of CTL from *T. parva*-immune twin animals conferred protection in naïve recipient animals when challenged with *T. parva*.^16^ Several CTL-inducing *T. parva* antigens have been identified in the past,^20,21^ with the Tp1 antigen^22,23^ inducing immune responses in at least 70% of BoLA-6*01301/01302 [A18/A18v]-positive cattle when vaccinated by ITM, indicating that this is an immunodominant antigen in these haplotypes (unpublished data).

Viral vectors are among the few delivery systems capable of inducing CD8^+^ T cells, with adenoviral and modified vaccinia virus vectors among the more widely used. These viral vectors were developed in the past years and several have shown promising results,^24,25^ either used as single injections, or in homologous or heterologous prime-boost vaccination regimens. They induced potent humoral and cellular immune responses against several pathogens, including *Plasmodium falciparum*,^26^ Ebola virus,^27^ influenza virus,^28^ HIV,^29^ tuberculosis,^25^ and rift-valley fever virus.^30^ In the case of *T. parva* vaccine development, previous attempts to induce schizont-specific CTL immunity in cattle with other recombinant viral-vectored vaccines showed promising immunogenicity, however, with poor immunity upon challenge.^20,31^

In this study, we evaluated a heterologous prime-boost regimen, using the Tp1 schizont antigen delivered by the human adenovirus serotype 5 (HAd5) as prime and modified vaccinia Ankara (MVA) as boost for induction of CD8^**+**^ cells and for protection. As studies have shown that the presence of a signal peptide sequence upstream of a start codon can modify or increase CTL responses due to changes in secretion and/or expression of the antigen,^32^ we also sought to compare the CD8^+^ T cell immune responses induced by HAd5 and MVA expressing the Tp1 antigen containing the tissue plasminogen activator (tPA) signal peptide (SP), the native Tp1 signal peptide or the mature Tp1 protein without any signal peptide, and assess if any of these constructs could confer protection towards a lethal challenge with the *T. parva* parasite. Furthermore, we have evaluated a panel of immune parameters for correlation with the level of parasitemia and with protection.

## Results

### Tp1 is expressed by both HAd5 and MVA vectors and efficiently processed and presented in infected cells

**Fig. 1:**
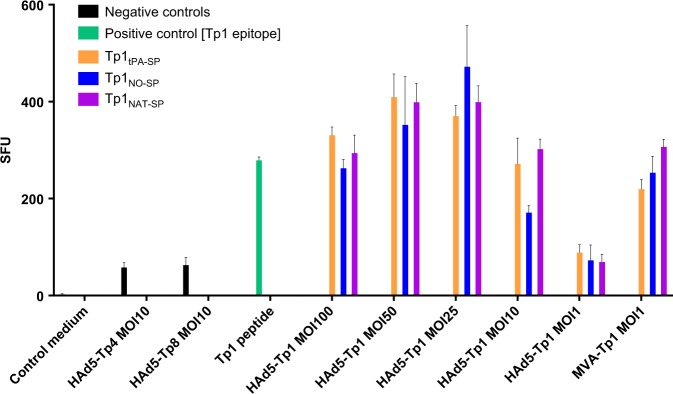
Expression and presentation of Tp1 by HAd5 and MVA vectors. IFN-γ-ELISpot assay performed with Tp1-specific CTL (BB007) and stimulated with the Tp1 peptide epitope, or with bovine fibroblast cells (BFC633) of the A18 haplotype infected with control constructs expressing irrelevant *T. parva* antigens Tp4 or Tp8, HAd5-Tp4 (MOI of 10) and HAd5-Tp8 (MOI of 10), or with HAd5-Tp1 (NO-SP, NAT-SP, and tPA-SP) at an MOI of 1, 10, 25, 50, 100, and MVA-Tp1 (NO-SP, NAT-SP, and tPA-SP) at an MOI of 1. Background levels are indicated as control medium. SFU spot-forming units

Utilization of adenovirus and MVA as viral vectors for antigen delivery relies on their ability to efficiently infect and express a transgene in inoculated tissues or antigen-presenting cells. We inserted the Tp1 gene with the three different signal peptide versions in HAd5 and MVA and tested the expression of the antigen in bovine fibroblast cells (BFC). Efficient generation of peptides by the immunoproteasome, translocation of peptides into the endoplasmic reticulum, and binding to the MHC class I molecules are important mechanisms for stimulation of T lymphocytes. To test this, BFC of the A18 haplotype were infected with HAd5 at multiplicity of infection (MOI) ranging from 1 to 100 and MVA at an MOI of 1, the latter due to the high cytotoxicity associated with this viral vector. An ELISpot was performed by coculturing the infected BFC and a Tp1-specific CTL line. The ELISpot revealed an equal level of presentation and stimulation of the CTL by the infected BFC for all constructs. A lower expression level of the Tp1 protein is observed for the MVA constructs, which is most probably due to the lower MOI used and the remaining cytotoxicity at this low MOI (Fig. 1).

In addition to this, both Vero and BFC cells were infected with HAd5 or MVA, lysed with radioimmunoprecipitation assay buffer and a western blot was performed with rabbit anti-Tp1 serum to evaluate Tp1 protein expression from the three different constructs. No marked differences in expression levels were observed between constructs possessing different signal peptides (data not shown). This showed that all constructs were functional in generating the Tp1 epitope and could therefore be tested in an in vivo experiment.

### All viral vectors induce strong Tp1-specific CD8^+^ and CD4^+^ T cell responses

**Fig. 2:**
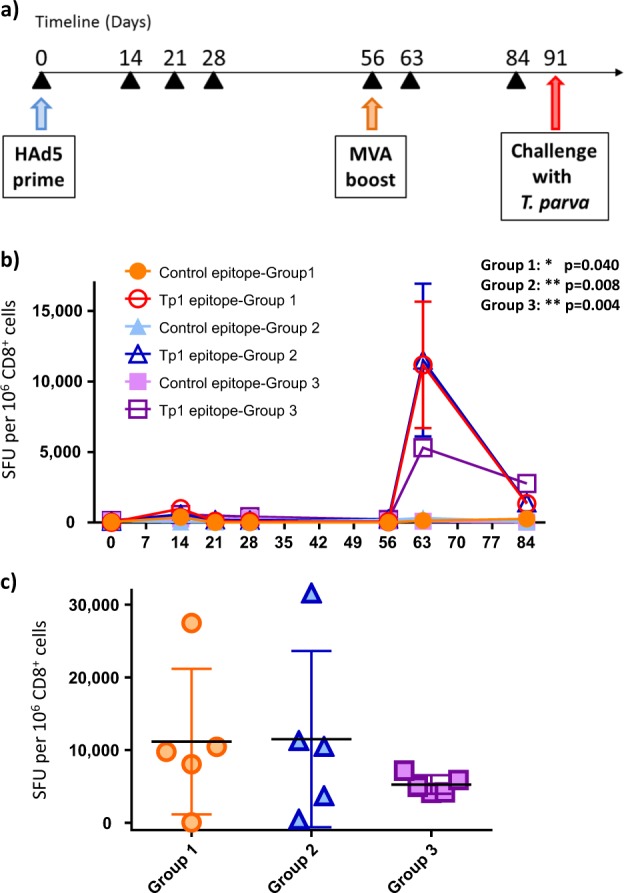
Induction of Tp1-specific response in ex vivo CD8^+^ T lymphocytes. **a** Timeline of the immunization and challenge experiment. Black arrows indicate time of sampling to perform ELISpot, flow cytometry, proliferation, and CTL assays. **b** IFN-γ ELISpot performed using CD8-purified cells at days 0, 14, 21, 28, 56, 63, and 84 post HAd5-Tp1 prime. The group average spot-forming units (SFU) and standard error mean (S.E.M.) are shown. Cells were exposed to irrelevant *T. parva* Tp2 epitope or Tp1 epitope. Group 1: HAd5/MVA-Tp1_tPA-SP_, Group 2: HAd5/MVA-Tp1_NO-SP_, Group 3: HAd5/MVA-Tp1_NAT-SP_. Statistical test for comparing Tp1 responses with control epitope responses 1-week post-MVA boost (day 63 post HAd5 prime): Mann–Whitney test (*p-*values: group 1 = 0.040, group 2 = 0.008, group 3 = 0.004). **c** IFN-γ ELISpot performed on CD8-purified cells 1-week post-MVA boost (day 63 post HAd5 prime); average SFU and standard deviation (S.D.) are shown. Orange circles: “group 1”, blue triangles: “group 2”, purple squares: “group 3”

Three groups of five cattle were inoculated intramuscularly in the mid-neck muscle with 2 × 10^9^ infectious units of HAd5 followed by intramuscular injection of 7.5 × 10^8^ plaque-forming units (pfu) of MVA into the muscle on the opposite side, 8 weeks post HAd5 prime for each of the three constructs (Fig. 2a). The Tp1-specific responses were monitored at several time points post HAd5 prime (Fig. 2a). First, a Tp1 epitope IFN-γ-ELISpot assay was conducted using ex vivo CD8-purified lymphocytes at days 0, 14, 21, 28, and 56 post HAd5 prime, as well as at day 7 and 28 post-MVA boost (63 and 84 post HAd5 prime). A slight increase in the average group IFN-γ production was observed 2 weeks post HAd5 prime after stimulation with the Tp1 peptide epitope, but this resolved back to background levels at day 21 (Fig. 2b). One week following MVA inoculation, a peak in the CD8^**+**^ Tp1-specific populations appeared, showing substantially boosted responses compared to days 0 and 14 post HAd5 prime (Fig. 2b) [all *p-*values <0.040]. When comparing the three groups, the first two groups (Tp1_tPA-SP_ and Tp1_NO-SP_) showed a similar range of CD8^**+**^ T cell response upon stimulation with the Tp1 epitope with means of 11,168 and 11,507 spot-forming units (SFU) per million (10^6^) CD8^+^ cells, respectively (Fig. 2c). However, a substantial variation in the response was observed, with one animal of the first group which did not respond to the known Tp1 epitope but did respond to the Tp1 peptide pool [data not shown], and with animals generating as low as 72 SFU per 10^6^ CD8^+^ cells up to 31,584 SFU per 10^6^ CD8^+^ cells in these first two groups. In the third group (Tp1_NAT-SP_), a lower mean of 5265 SFU per 10^6^ CD8^+^ cells was observed, with much less variation in the responses between animals, ranging from 4132 to 7168 SFU per 10^6^ CD8^+^ cells (Fig. 2c). However, no statistically significant differences were observed between the groups [*p-*value = 0.505].

**Fig. 3:**
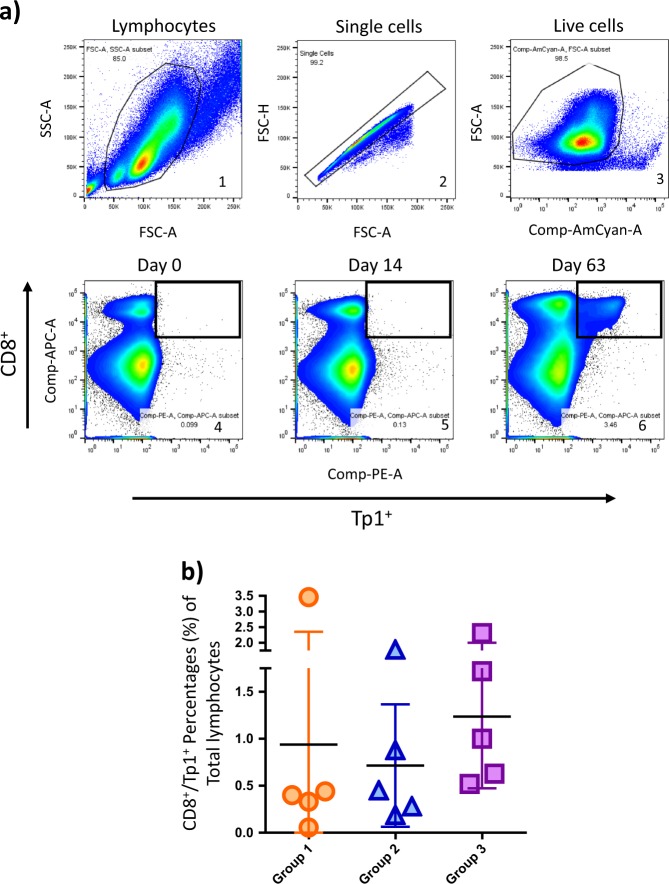
Flow cytometry analysis of Tp1-specific CD8^+^ lymphocytes. **a** Example of sample gating of lymphocytes (gate 1), single cells (gate 2), live cells (gate 3), Tp1^+^/CD8^+^ population at day of priming with HAd5 (gate 4), Tp1^+^/CD8^+^ population 14 days post adenovirus-prime (gate 5), Tp1^+^/CD8^+^ population 7 days post-MVA boost, or 63 days post HAd5 prime (gate 6). **b** Average percentage standard deviation (S.D.) are shown for Tp1^+^/CD8^+^ lymphocytes from each group at 7 days post-MVA boost (63 days post HAd5 prime). Group 1: HAd5/MVA-Tp1_tPA-SP_ (orange circles), Group 2: HAd5/MVA-Tp1_NO-SP_ (blue triangles), Group 3: HAd5/MVA-Tp1_NAT-SP_ (purple squares)

In parallel with the ELISpot assay, evaluation of the Tp1-specific responses was conducted by flow cytometry using Tp1-BoLA-6*01302 peptide-MHC class I tetramers as described in a previous study.^33^ PBMC were stained with anti-bovine CD8 antibody concomitantly with Tp1-BoLA-6*01302 tetramers and the percentages of Tp1-specific CD8^+^ cells were evaluated. Analyses were performed by gating on lymphocytes, single cells, live fraction population, and double-positive Tp1^**+**^/CD8^**+**^ lymphocytes (Fig. 3a). This revealed percentages in the range of 0–3.46% of PBMC, with averages ranging from 0.72–1.24% which is comparable to the percentage of Tp1^+^/CD8^+^ lymphocyte population generated during an ITM immunization regimen.^33^ No statistically significant differences were observed between the groups [*p-*value = 0.231] (Fig. 3b). All animals had measurable responses by flow cytometry except for one animal, which did not show any response to the known Tp1 epitope as initially observed in the ELISpot assay (Fig. 2). In conclusion, the vaccination seemed to induce robust Tp1 epitope-specific CD8 responses.

Previous reports have shown the potency of the HAd5/MVA vaccination system for induction of strong CD4 responses.^34^ Even though Tp1 is only known for its ability to induce CD8^**+**^ T cells, we also evaluated the induction of CD4^**+**^ T cells. This was included to get an indication of the ability of the prime/boost regimen to induce CD4^**+**^ T cells in cattle but there was also a possibility that Tp1 itself could be a good CD4 antigen, as this had not previously been tested. Slight proliferations of total PBMC and purified CD4^**+**^ T lymphocytes were measured at 14 days post HAd5 prime (data not shown), upon exposure to the Tp1 peptide pool or recombinant full-length Tp1 protein. In accordance to what was observed for the CD8^**+**^ T cell response, robust proliferation of PBMC (data not shown) and CD4^**+**^ T lymphocytes (Supplemental Fig. 1) could be observed 7 days post-MVA boost in all groups. This shows that HAd5/MVA can potentially be a good system to generate CD4^**+**^ responses towards *T. parva*-specific T helper antigens in cattle, and it is established knowledge that CD4^**+**^ T cells are required for priming of both CD8^**+**^ T cells and B cells, in most systems (reviewed in ref.^35^).

### Generation of effector and central memory lymphocytes

**Fig. 4:**
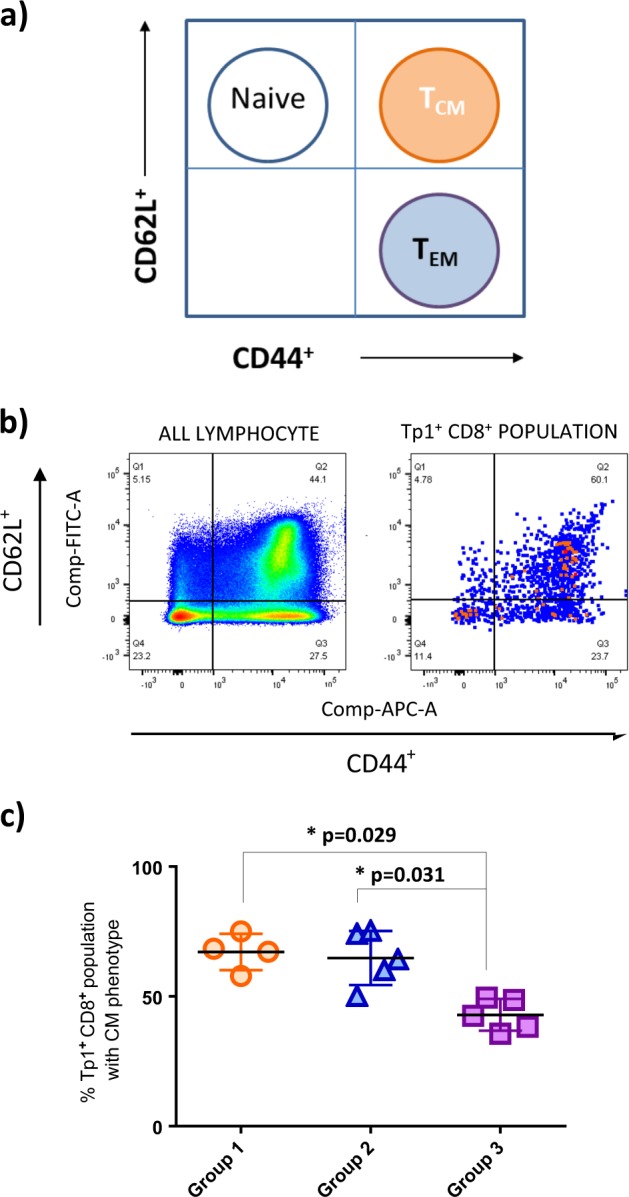
Induction of central memory Tp1^+^/CD8^+^ cells. **a** Schematic plot of T central memory (T_CM_) and T effector/effector memory (T_EFF/_T_EM_) lymphocytes by staining for activation (CD44) and migration (CD62L) markers.^68^ **b** A live example of pseudo color plots of CD44 and CD62L expression from total lymphocytes (left) and from Tp1^**+**^/CD8^**+**^-gated cells from one animal in group 1, 7 days post-MVA boost (right). **c** Percentage of T_CM_ lymphocytes from the Tp1^+^/CD8^+^ population 7 days post-MVA boost (63 days post HAd5 prime). Group 1: HAd5/MVA-Tp1_tPA-SP_ (orange circles), Group 2: HAd5/MVA-Tp1_NO-SP_ (blue triangles), Group 3: HAd5/MVA-Tp1_NAT-SP_ (purple squares). Statistical analysis: Kruskal–Wallis test with multiple comparison [overall *p-*value = 0.003]

Several studies have highlighted the relationship between the presence of central memory T cells (T_CM_) and protection from disease. T_CM_ are located in lymphoid organs and can proliferate and become activated more rapidly upon recognition of a specific epitope^35^ compared to effector memory (EM) cells, which are present in peripheral tissues and expand less rapidly upon antigen recognition. Several markers are associated with CM and EM activation. Among available markers in bovine immunology, we used L-selectin (CD62L) and CD44 as markers of migration and activation respectively since previous studies have shown that central memory cells express both of these markers^36,37^ (Fig. 4a). These markers have also successfully been used in bovine immunology studies.^38–40^ We analyzed the percentage of Tp1^**+**^/CD8^**+**^/CD44^**+**^/CD62L^**+**^ in each animal of the three different groups. The animal with no measurable Tp1^**+**^ population was not included in the analysis. Analyzing these populations indicated that T_CM_ are present in higher frequencies in the first two groups than the third group (*p-*values of 0.029 and 0.031, respectively) with averages of 67.13% and 64.93% for groups 1 and 2 (Tp1_tPA-SP_ and Tp1_NO-SP_), whereas group 3 (Tp1_NAT-SP_) shows a lower percentage of T_**CM**_ cells with an average of 44.73% (Fig. 4b, c).

### Generation of CTL by HAd5/MVA-Tp1 vaccination

**Fig. 5:**
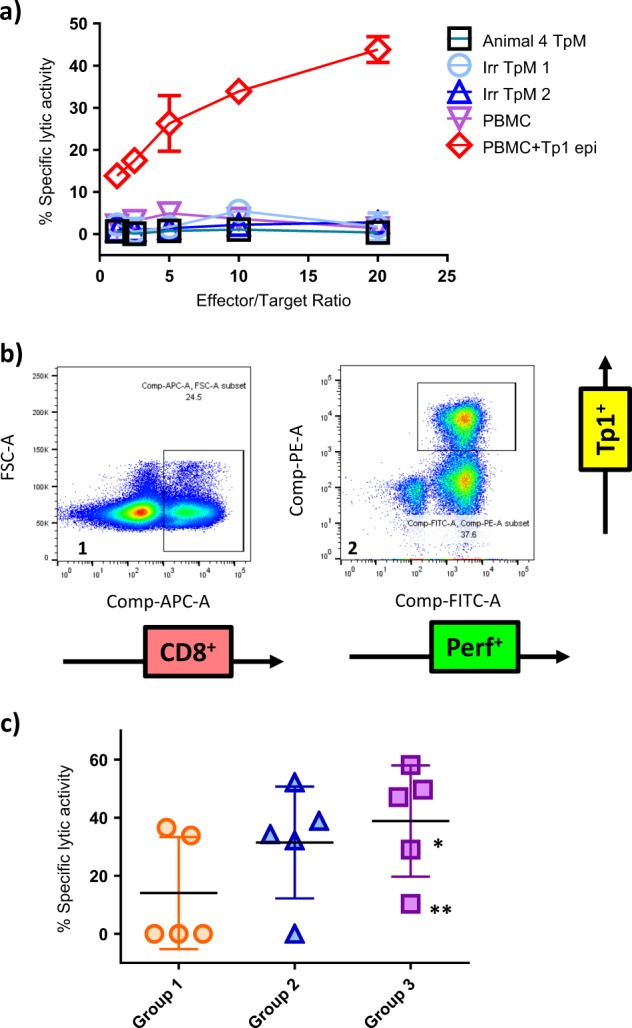
Cytotoxicity of CTL generated 4 weeks post-MVA boost. **a** Example of the CTL data generated. Bulks were generated from each animal and assays were performed in twofold dilutions of the CTL culture as indicated on the graph. Negative controls included two irrelevant TpM and un-pulsed PBMC. **b** Example of a Tp1^+^/perforin^+^ staining (gate 2) on the total CD8^+^ population (gate 1) from the bulk CTL generated from animal 4 of group 1. **c** Percentage of specific CTL lysis of Tp1-pulsed autologous PBMC at 10:1 effector: target (E:T) ratio for each group at 4 weeks post-MVA boost, and following three rounds of stimulation with Tp1 peptide pool. Statistical analysis: Kruskal–Wallis test with multiple comparison [*p-*value = 0.179]. Group 1: HAd5/MVA-Tp1_tPA-SP_ (orange circles), Group 2: HAd5/MVA-Tp1_NO-SP_ (blue triangles), Group 3: HAd5/MVA-Tp1_NAT-SP_ (purple squares). *E:T ratio of 2:1; **E:T ratio of 5:1

To test if HAd5/MVA prime-boost regimen induced CD8^**+**^ T cells with lytic activity, we generated two bulk lines from each animal, one re-stimulated with autologous *T. parva* Muguga-infected lymphocytes (TpM) and the other with a pool of 16-mer peptides, overlapping by 12 amino acids, covering the full-length Tp1 antigen (Tp1_pool_), which includes the Tp1 epitope. We observed that the lines generated by the Tp1_pool_-stimulated cells lysed Tp1 epitope-pulsed autologous PBMC, but not autologous TpM (example shown in Fig. 5a). However, none of the lines generated by coculturing with autologous TpM lysed autologous TpM, even though there was robust recall of Tp1-specific CD8^**+**^ lymphocytes in some of the CTL bulks as measured by tetramer staining (data not shown). Two out of 15 animals generated CTL in the autologous TpM-stimulated bulk, which had detectable lytic activities towards Tp1_epitope_-pulsed autologous PBMC (data not shown). The Tp1-positive cells were found to be perforin-positive in all the bulk CTL that were generated (example shown in Fig. 5b), indicating proper activation. In summary, we observed that 11 out of 15 animals had generated CTL from the Tp1_pool_-stimulated bulk lines, which had detectable lytic activities using Tp1 epitope-pulsed PBMC as target cells (Fig. 5c).

### Inoculation of cattle with HAd5/MVA-Tp1 confers partial protection at group level against a lethal challenge with *T. parva*

**Fig. 6:**
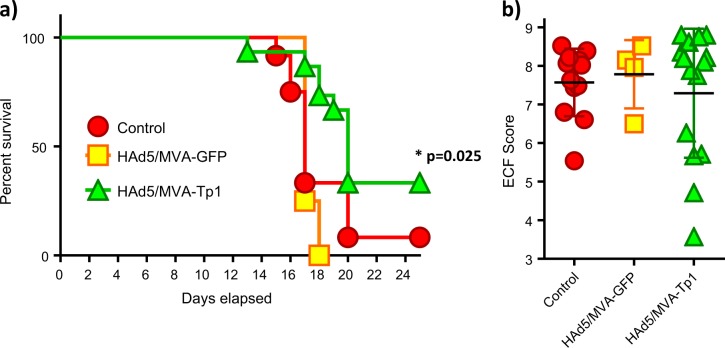
Survival curve and ECF indices following a lethal *T. parva* challenge. **a** Kaplan–Meier survival curve of the control animals (red), the HAd5/MVA-GFP control animals (yellow), and the HAd5/MVA-Tp1-immunized animals (green). Statistical analysis: Mantel–Cox test [*p-*value between HAd5/MVA-Tp1 and control: 0.025; *p-*value between HAd5/MVA-Tp1 and control plus HAd5/MVA-GFP as a single control group: 0.010]. **b** ECF indices. The animals of the control group are depicted as red circles; the animals of the control HAd5/MVA-GFP group are depicted as orange/yellow squares and the immunized animals are depicted as green triangles. GFP green-fluorescent protein. Statistical analysis: Kruskal–Wallis test with multiple comparison [*p-*value = 0.865]

To evaluate the protective capacity of the experimental vaccine, animals were re-tagged (blinded experiment) and challenged with a lethal dose 100% (LD100) of *T. parva* 5 weeks post-MVA boost (Fig. 2a). All Tp1-immunized animals were considered as one group since there were no statistical difference in the immune responses between the groups immunized with the three different Tp1 constructs. Two control groups, one which was inoculated with HAd5/MVA-GFP and another non-immunized group were challenged in parallel. Animals were injected subcutaneously with a 1:20 dilution of the *T. parva* Muguga strain (stabilate 3087). Animals were monitored daily and an ECF index was generated by collecting thirteen parameters including onset of fever, onset of schizonts and piroplasms as described previously.^41^ Of the 15 animals inoculated with HAd5/MVA-Tp1, 5 animals were able to control the infection (Fig. 6a). One animal controlled the infection in the non-treated control group but none in the other control group, the HAd5/MVA-GFP inoculated group. A statistically significant difference was observed between the control animals and the HAd5/MVA-Tp1-immunized animals when comparing the survival Kaplan–Meier curve (Mantel–Cox, *p-*value: 0.025, and 0.010 if both control groups are combined). Evaluating the ECF index, control animals had ECF indices ranging from 5.54 to 8.52 whereas HAd5/MVA-Tp1-immunized animals had an ECF index ranging from 3.57 to 8.80 with four animals developing an ECF index below 6, indicating they were mild and mild/moderate reactors (Fig. 6b) and immune to ECF.

**Fig. 7:**
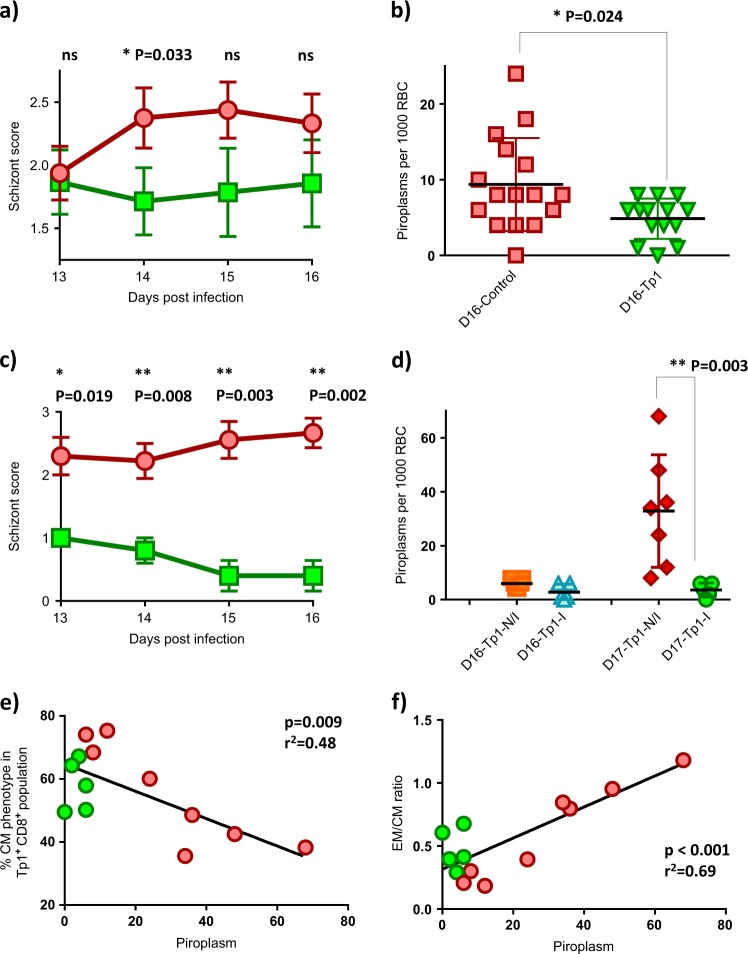
Parasitemia in relation to Tp1^+^ CD8^+^ central memory lymphocytes. **a** Schizont scores with standard error mean (S.E.M.) in contralateral lymph nodes from days 13 to 16 post challenge. Red circles: All 16 control animals [non-immunized and HAd5/MVA-GFP-inoculated animals]. Green squares: All 15 HAd5/MVA-Tp1-inoculated animals. Statistical analysis: Mann–Whitney, *p* = 0.033. **b** Numbers of piroplasm per 1000 red blood cells (RBC) at day 16 post *T. parva* challenge. Red squares: all 16 control animals [non-immunized and HAd5/MVA-GFP-inoculated animals], green triangles: all 14 HAd5/MVA-Tp1 remaining inoculated animals [one animal was euthanized at day 13 before developing piroplasm]. Statistical analysis: Mann–Whitney, *p* = 0.024. **c** Schizont scores in contralateral lymph nodes. Red circles: all 10 non-immune HAd5/MVA-Tp1-inoculated animals, green squares: all 5 immune HAd5/MVA-Tp1-inoculated animals. Statistical analysis: Mann–Whitney, overall *p-*value <0.020. **d** Numbers of piroplasm per 1000 red blood cells (RBC) at days 16 and 17 post *T. parva* challenge. Orange squares: nine remaining non-immune HAd5/MVA-Tp1-inoculated animals at day 16 post challenge, blue triangles: five immune HAd5/MVA-Tp1-inoculated animals at day 16 post challenge, red diamonds: seven remaining non-immune HAd5/MVA-Tp1-inoculated animals at day 17 post challenge, green circles: five immune HAd5/MVA-Tp1-inoculated animals at day 17 post challenge. Statistical analysis: Mann–Whitney, *p* = 0.003. **e** Percent (%) Tp1^**+**^/CD8^**+**^ lymphocytes with central memory (CM) phenotype in relation to levels of piroplasm. Green circles: immune animals, red circles: non-immune animals. Statistical analysis: linear regression, *p* = 0.009, *r*^2^ = 0.48. **f** % Effector memory (EM)/% central memory (CM) ratio in relation to levels of piroplasm. Green circles: immune animals, red circles: non-immune animals. Statistical analysis: linear regression, *p* < 0.001, *r*^2^ = 0.69

Among all 13 parameters collected for the calculation of the ECF index, we have observed that the level of parasitemia in the blood was significantly lower (*p-*value: 0.033 and 0.024 for schizonts at day 14 post infection and piroplasm, respectively) in all immunized animals at the onset of schizonts in the contralateral lymph node and of piroplasmosis (at day 16 post infection) in the blood (Fig. 7a, b). Within the HAd5/MVA-Tp1-immunized group, there is a clear distinction in the parasite load between the immune and non-immune animals with the former showing significantly lower number of parasites (Fig. 7c, d, all *p-*value <0.020 and 0.003 for the schizonts and piroplasm, respectively). Parasitemia, and especially piroplasmosis, is an important parameter defining the ECF index since 9 out of 13 parameters are based on parasite measurements. This also explains the reason why some authors have used the piroplasm value as the main biological parameter to categorize animals according to ECF severity as developed in the Schetters scoring method.^42^ Interestingly, among all immunological parameters measured, a higher percentage of the Tp1^**+**^/CD8^**+**^ cell population displaying a central memory phenotype correlated (*p-*value = 0.009, *r*^2^ = 0.48) with a lower level of piroplasm (Fig. 7e). The correlation is even more evident when comparing the CD8^**+**^ EM/CM ratio^43^ in relation to piroplasm levels (*p-*value = 0.004, *r*^2^ = 0.69) (Fig. 7f). This suggests that the Tp1-specific CTL response has a positive effect in controlling the overall level of parasites in the blood and that central memory cells could be important for an efficient control of parasites in infected animals. This correlation would need to be further confirmed in future experiments.

## Discussion

The ITM, using the Muguga cocktail vaccine, is an efficient way of vaccinating against ECF and it has successfully been utilized in, e.g. Tanzania, for protection of cattle.^44,45^ Substantial evidence indicates that cytotoxic CD8^+^ T lymphocytes induced by the ITM vaccine are crucial for protecting the animals against the *T. parva* parasite. Therefore, induction of CD8^**+**^ T lymphocytes is an important parameter to consider in the development of subunit vaccines towards ECF. Here we report for the first time the use of HAd5/MVA prime-boost regimen for delivering a *T. parva* CD8^**+**^ antigen. Upon boosting with MVA-Tp1, we measured a Tp1 epitope-specific CD8^**+**^ response in 93% of animals and observed a level of protection of 33% upon a lethal challenge compared to 6% (1 out of 16) of control animals surviving the challenge. When considering the 14 Tp1 epitope-responding animals only, the level of protection was 36%. A more objective way to compare these results with previous ECF immunization studies is to use the ECF index,^41^ which define mild or mild/moderate reactor animals as having an ECF index below 6 and moderate/severe and severe reactor animals with an ECF index of 6 and above. Using this criterion, we observe that 6% of the animals are mild/moderate reactors in the combined control group whereas 27% are mild or mild/moderate reactors in the vaccinated group. In a previous study using DNA or canarypox virus prime followed by MVA boost, with a panel of *T. parva* CTL antigens, CD8^**+**^ responses were induced in 79% of animals but only 8% were mild or mild/moderate reactors in the vaccinated group when considering the ECF indices.^20^ Therefore, a clear improvement of almost three-fold was obtained in this present HAd5/MVA study, compared to the previous immunization protocol. Further, it is important to note that the challenge dose used in this study does not reflect the exposure level of the parasite in natural conditions, which is closer to an LD70 on average, estimated from the lethality found in field-settings on herd level.^46,47^ A challenge with a more natural level of sporozoites would probably increase the level of protection induced by this vaccination strategy.

The presence or absence of signal peptides has been reported to have a great impact on the expression and presentation of antigens^32^ and hence immune responses. When comparing immune responses in the three groups, slightly higher levels of specific lysis of the Tp1-pulsed PBMC could be observed from the group of animals receiving the Tp1 with the native signal peptide (Group 3), but differences were not statistically significant. Group 3 also had a lower, but more evenly distributed response towards the Tp1 epitope in the ELISpot assay and slightly higher percentages of Tp1-specific cells as measured by tetramer analysis, however, with lower central memory phenotype. This is unlikely to be due to differences in expression levels, presentation levels, or export of Tp1, as there were no differences in these parameters in vitro between Tp1 constructs (Fig. 1 and data not shown). Nevertheless, it would require substantially more animals per group to establish significant differences between the three animal groups. Hence, in this study, we have treated the three immunized groups as one.

ITM vaccination confers a protection level close to 100%,^48^ whereas 27% was obtained in this study, after retrieving the 6% survival rate in the combined control groups. Reasons for this could be differences in the magnitude or the quality of the CTL response generated by the two regimens. However, animals immunized by ITM develop similar levels of Tp1-specific CD8^**+**^ T cells as seen in the HAd5/MVA immunized animals, when assessed by ex vivo ELISpot and tetramer analysis^33^ (and unpublished data), indicating that the differences in protection using the two regimens are not attributed to quantitative differences in Tp1-specific cytotoxic T cells. A more likely explanation is that the observed differences are simply due to the number of antigens used in the two systems. The parasite genome consists of more than 4000 genes, making it likely that several proteins will function as antigens on individual infected cells when the ITM regimen is used and hence the CTL will kill them more easily. The reflection of this may be a lower protection level of the HAd5/MVA-immunized animals and lack of killing of infected cells in vitro, as observed in this study, even though we could measure specific CTL activity on autologous TpM for a larger fraction of the animals in a subsequent experiment (data not shown, manuscript in preparation). However, it has previously been suggested that there is a marked immunodominance operating in animals immunized using ITM,^22,49–51^ but these studies were in most cases performed using MHC class I homozygous animals, which will skew the response towards certain epitopes. Subdominant CTL responses have been shown to exist in immune responses to *T. parva*^52^ and this could contribute to the overall cytotoxic activity towards *T.* parva-infected lymphocytes. Several CTL antigens have previously been identified,^20^ of which some were also good CD4^**+**^ T cell inducers (Ivan Morrison, personal communication). The latter is important as CD4^**+**^ lymphocytes are necessary for priming and recalling of CD8^**+**^ memory cells in most systems (reviewed in ref. ^35^). It would be interesting to include other CTL or CD4^**+**^ antigens in the regimen in the future, to see if the protection level improves.

Another explanation for the differences in protection levels obtained by ITM and HAd5/MVA could be due to the qualities of the Tp1-specific CD8^+^ T cells in the two regimens, e.g., it is known that the level of antigen can influence the TCR affinity/avidity,^53^ and the level of Tp1 expression in vivo could differ among the two regimens. Further, *T. parva* is known to modulate the immune response by increasing IL-10.^54,55^ Interestingly, a recent study indicated that this cytokine environment could guide a better maturation of CD8^**+**^ T lymphocytes to the memory phenotype,^56^ and this might be suboptimal in HAd5/MVA-immunized animals. Other parameters that can explain suboptimal efficiency of the CTL is the diversity of the T cell receptors selected during the response towards a single antigen, and polyfunctionality of the Tp1-specific T cells. These parameters have previously been shown to correlate positively with a better protection upon challenge^57^ for other diseases. Several of the mentioned parameters that can influence the quality of T cells are currently being assessed at ILRI in a parallel experiment (data not shown, manuscript in preparation).

The success of the ITM vaccine resides in its capacity to induce long-lived immunity towards *T. parva*. A crucial feature of vaccines that induce life-long protection is their ability to induce memory cells, which can respond to antigens previously encountered in infection or vaccination. Several studies have indicated the important role of both effector and central memory CD8^**+**^ lymphocytes in providing protection and controlling parasitemia or viremia in infected hosts. Studies have shown that the human immunodeficiency virus (HIV) long-terminal non-progressors (elite controllers) develop a high percentage of Gag-specific CD8^**+**^ central memory lymphocytes compared to progressors,^58^ and this correlates positively with lower viral loads.^59^ Similarly, experiments conducted in mice inoculated with *Plasmodium* sp. parasites demonstrated a role of CD8^**+**^ memory T cells in conferring protection and indicated a correlation between memory cells and lower parasitemia.^60^ In this study, we observed a negative correlation of the Tp1^**+**^/CD8^**+**^-specific central memory cells and the parasitemia (numbers of piroplasms in whole blood). This agrees with previous reports demonstrating that adenovirus vectors can be good inducers of central memory CD8^**+**^ T cells.^61^ This also indicates that central memory CD8^**+**^ lymphocytes are key players in providing control of *T. parva* parasites upon challenge.

In summary, we have demonstrated that the HAd5/MVA prime-boost immunization regimen has the capacity to induce CTL directed to the Tp1 antigen from *T. parva* and that it confers partial protection against a lethal challenge with the parasite. This is the first report showing that just one *T. parva* CTL antigen can induce partial protection towards a *T. parva* infection. These results suggest that the HAd5/MVA vectors are potent inducers of CD8^**+**^ memory T lymphocytes in cattle and that this delivery system can be used for down-selection of potential pathogen antigens to include in a subunit vaccine. It is conceivable that more antigens are required for obtaining full protection and this will eventually also be required for obtaining protection in animals that have not been BoLA-preselected and for covering the variety of *Theileria parva* strains circulating in the region.

## Materials and methods

### Generation of human adenovirus serotype 5 and MVA viral vectors expressing Tp1

The gene encoding the mature Tp1 protein^20^ from *Theileria parva* Muguga isolate [stabilate 3087] without a signal peptide [Tp1_NO-SP_] (mature 524 amino acids protein), with the tPA signal peptide [Tp1_tPA-SP_], or the full-length Tp1 protein containing the native signal peptide (MRVKKVLLYTLPVVGILLA) [Tp1_NAT-SP_] was inserted in non-replicative human adenovirus serotype 5 (HAd5) in place of the E1A and E1B adenoviral genes under the CMV promoter and in Modified Vaccinia virus Ankara (MVA) under the p7.5 promoter as previously described.^62–64^ Vaccine doses were derived from a single batch preparation manufactured at the Viral Vector Core Facility, Jenner Institute, Oxford, UK from seed stocks using specific pathogen-free CEF and T-Rex 293 A cell lines for MVA and HAd5, respectively, in certified pathogen-free media. HAd5 and MVA vectors expressing green-fluorescent protein (GFP) in place of the Tp1 protein were manufactured in parallel to a similar viral titer and used for control vaccination.

### In vitro HAd5/MVA-Tp1 expression and presentation assay

For Tp1 protein expression, and Tp1 epitope processing and presentation, bovine fibroblasts of the A18 haplotype (in-house cell line BFC633) were infected with HAd5-Tp1_tPA-SP_, HAd5-Tp1_NAT-SP_, or HAd5-Tp1_NO-SP_ at a multiplicity of infection (MOI) ranging from 1 to 100, and with MVA-Tp1_tPA-SP_, MVA-Tp1_NAT-SP_, or MVA-Tp1_NO-SP_ at an MOI of 1. Fibroblasts were collected 24 h post infection and exposed to 2.5 × 10^4^ cells/well of an in-house A18 Tp1-specific CTL line (BB007) and activation was measured with an ELISpot assay as described below. Medium alone, HAd5 expressing irrelevant *T. parva* antigens Tp4 and Tp8 restricted by another BoLA class I molecule (HAd5-Tp4 and HAd5-Tp8) at an MOI of 10 were used as negative controls, and Tp1 peptide epitope alone was used as a positive control.

### Cattle, viral inoculation, and challenge

Fifteen Holstein/Friesians, 7- to 13-month-old male cattle, of the A18v (BoLA-6*01302) haplotype, were divided into three groups of five animals. Group 1 received the HAd5/MVA-Tp1_tPA-SP_, group 2 was inoculated with HAd5/MVA-Tp1_NO-SP_, and group 3 was inoculated with HAd5/MVA-Tp1_NAT-SP_. Each animal received 2 × 10^9^ infectious units (IU) of HAd5 and 7.5 × 10^8^ pfu of MVA, 56 days post HAd5 prime, via the intramuscular route in the neck muscle. For challenge, a dilution of 1:20 of the *T. parva* Muguga stock 3087 (equivalent to an LD100 dose) [data not shown] was injected subcutaneously in-front and below the ear over the parotid lymph node 5 weeks post-MVA boost. The experiment was blinded by re-tagging animals before challenge. Clinical and biological parameters were monitored, and a Rowlands’ ECF index was calculated as described.^41^ A higher ECF index indicates a higher susceptibility. Briefly, an ECF index of 0–0.99 = non-reactor, an ECF index of 1–3.99 = mild reactor, an ECF index of 4–5.99 = mild/moderate reactor, an ECF index of 6–7.99 = moderate/severe reactor, and an ECF index of 8–10 = severe reactor. Schizont scores from blood and lymph node smears were evaluated as follows: 0 = no schizonts, 1 = 1 schizont per every 5 microscopy field, 2 = 1–2 schizonts per field, 3 = 3 or more schizonts per field. Piroplasm were calculated as number per 1000 red blood cells (RBC). Animals were euthanized when reaching clinical end-points as indicated by ILRI standard operating procedures. A group of 12 animals not inoculated with the viral vectors and 4 animals inoculated with HAd5-GFP/MVA-GFP were used as negative controls. The animal experiment received approval by ILRI IACUC committee (ref. no. 2014.13).

### Flow cytometry

Tp1-BoLA-6*01302 peptide-MHC class I tetramer was generated as previously described.^33,65,66^ Whole PBMC were purified on Ficoll-Paque as previously described.^33^ Cells were stained with 10 µl of Tp1-BoLA-6*01302 peptide-MHC class I tetramer and antibodies against CD8 (IgG1, ILA51), CD62L (directly labeled-FITC; AbD Serotec, mouse anti-human CD62L, MCA1076F), CD44 (ILA108) at dilutions of 1:250 and human anti-perforin-FITC (BD Pharmingen, cat # 556577, ready-to-use). Primary antibodies were labeled with secondary anti-IgG1-PerCP (Becton Dickinson, cat # 340272) at 1 µl/sample or anti-IgG1-APC (BioLegend, clone RMG1-1, cat#406610) and anti-IgG2a-APC (BioLegend, clone RMG2a-62, cat # 407110) at a dilution of 1:200 [25 µl per sample]. All samples were stained with Fixable Viability Stain 450 (BD Horizon, cat # 562247). Staining was done in PBS–0.5% BSA except for intracellular staining with anti-perforin where cells were incubated and stained in PBS–0.1% Saponin–10% FBS. Samples were analyzed on a BD FACS Canto II flow cytometer and data were analyzed with FlowJo. Compensation controls for PE, PerCP, FITC, Pacific Blue or AmCyan, and APC were included for automatic compensation by the FACS DIVA software. For the analysis of ex vivo cells, 500,000 events in the lymphocytes gate were acquired.

### BoLA class I typing

A first screening for A18 and A18v haplotypes was performed on a number of Holstein/Friesian cattle originating from Nyeri, Kenya, using ILRI anti-BoLA antibodies (ILA35 and B4/18) and flow cytometry analysis. Positive A18 haplotypes were confirmed by ELISpot assay using Tp1 peptide pulsing of PBMC from sampled animals and matched A18v Tp1-specific CTL lines. Further confirmation was performed by SSP-PCR followed by RFLP and sequencing as previously described.^67^

### IFN-gamma (γ) ELISpot assay

Interferon (IFN)-gamma (γ) enzyme-linked immunospot (ELISpot) assay was performed as previously described.^33^ Briefly, CD8^**+**^ cells from whole Ficoll-purified PBMC were isolated using the MACS Cell Separation system (with anti-mouse IgG MicroBeads, Miltenyi Biotec, cat # 130-048-401) following the manufacturer’s instructions. A monoclonal anti-bovine IFNγ antibody (Serotec, Oxford, UK, cat. no. MCA1783) was incubated overnight at 4 °C on ELISpot plates (Millipore, cat # MAIPN4550, Billerica, MA, USA) and then blocked with RPMI containing 10% heat-inactivated FBS for 2 h at 37 °C. Peptides were added at 1 μM concentration and CD8-positive cells at varying concentrations ranging from 3.125 × 10^4^ to 2.5 × 10^5^ cells per well. The plates were incubated at 37 °C for 20 h. Release of IFN-γ was monitored with primary rabbit polyclonal anti-bovine IFN-γ antibody (Sigma-Aldrich, St. Louis, MO, USA) and secondary AP-conjugated monoclonal anti-rabbit IgG (Sigma-Aldrich, St. Louis, MO, USA, cat # A2556). Development of plates was done by addition of the substrate solution Sigma Fast (BCIP/NBT, Sigma-Aldrich, cat # B5655-25TAB, St. Louis, MO, USA).

### CD4/PBMC proliferation assay

Whole PBMC were purified on Ficoll-Paque. CD4^**+**^ T cells were labeled with anti-CD4 [ILA11], purified by the MACS Cell Separation system (with anti-mouse IgG MicroBeads, Miltenyi Biotec, cat # 130-048-401), and were cultured at different dilutions (ranging from 1 × 10^3^ to 5 × 10^4^ cells per ml) in the presence of 4 µg/ml of recombinant Tp1 protein (EXPRES^**2**^ION Biotechnologies, Horsholm, Denmark) or 1 µg/ml of Tp1 peptide pool [16-mers overlapping by 12 amino acids covering the whole protein including the native signal peptide] (Mimotopes). Irrelevant Tp2 peptide pool (1 µg/ml) and ovalbumin (Sigma, cat# A5253-250G) at 4 µg/ml was used as a negative control and ConA as a positive control. Proliferation was monitored by labeling with thymidine-H^3^ (0.5 µCi per well, American Radiolabeled Chemicals, St-Louis, MO, cat # ART 0178 J) four days post culturing. Counts per minutes (CPM) were measured in a TopCounter (PerkinElmer, Waltham, MA, USA). Fold increase was calculated by dividing the CPM of the stimulated wells by the CPM of the cells with media alone at day 63 post HAd5 priming. CPM values of control media was between 800 and 7000.

### Cytotoxicity assay

Blood was collected from each animal 5 weeks post-MVA boost (day 91 post HAd5 prime) and PBMC were purified on Ficoll-Paque. PBMC were then cultured with irradiated *Theileria parva* transformed (infected) autologous lymphocytes [TpM] at a 10:1 ratio or with Tp1 peptide pool [1 µM] in RPMI with 10% FBS to generate polyclonal bulk CTL lines. Following three rounds of stimulation (1-week interval between re-stimulations), CD8^**+**^ T cell cytotoxicity assay was performed as described^66^ and the full protocol can be viewed in ref. ^65^ Briefly, a standard 4 h-release assay using ^51^Cr-labeled target cells was used. ^51^Cr was obtained from American Radiolabeled Chemicals, Inc., St. Louis, MO, USA. Supernatants were counted using Lumaplates (PerkinElmer, Waltham, MA, USA) in a TopCounter (PerkinElmer, Waltham, MA, USA). The cytotoxicity was calculated as the: (experimental release minus spontaneous release/total release minus spontaneous release). Target cells were either *T. parva* Muguga 3087-infected autologous PBMC or Tp1 epitope (^**214**^VGYPKVKEEML_**224**_)-pulsed autologous PBMC. Mismatched TpM and non-pulsed PBMC served as negative controls. Peptide pulsing was performed before ^51^Cr-labeling using 1 μM of the desired peptide.

### Statistical analysis

Statistical analysis of data was performed with the GraphPad Prism version 6 software. When comparing two independent groups, the non-parametric Mann–Whitney test was used. When comparing three groups, a Kruskal–Wallis test with post-hoc multiple comparisons [Dunn’s test] was used. To assess the animal survival, a Kaplan–Meier curve was fitted and Mantel–Cox test was used to evaluate significance. To evaluate correlation of central memory CD8^**+**^ cells with piroplasm a linear regression was performed.

### Data availability

The corresponding authors declare that the data supporting the findings of this study are available upon reasonable request.

## Electronic supplementary material

Supplementary figure 1

## Electronic supplementary material

**Supplementary information** accompanies the paper on the *npj Vaccines* website (10.1038/s41541-018-0073-5).
