# Supplementary material for: An Ad/MVA vectored Theileria parva antigen induces schizont-specific CD8+ central memory T cells and confers partial protection against a lethal challenge
Source: NPJ Vaccines. 2018 Sep 11;3:35. doi: 10.1038/s41541-018-0073-5 (PMC6134044; doi:10.1038/s41541-018-0073-5)
Supplement: Supplementary file 1 — Supplementary figure 1 [file 41541_2018_73_MOESM1_ESM.pdf]

Supplementary figure 1

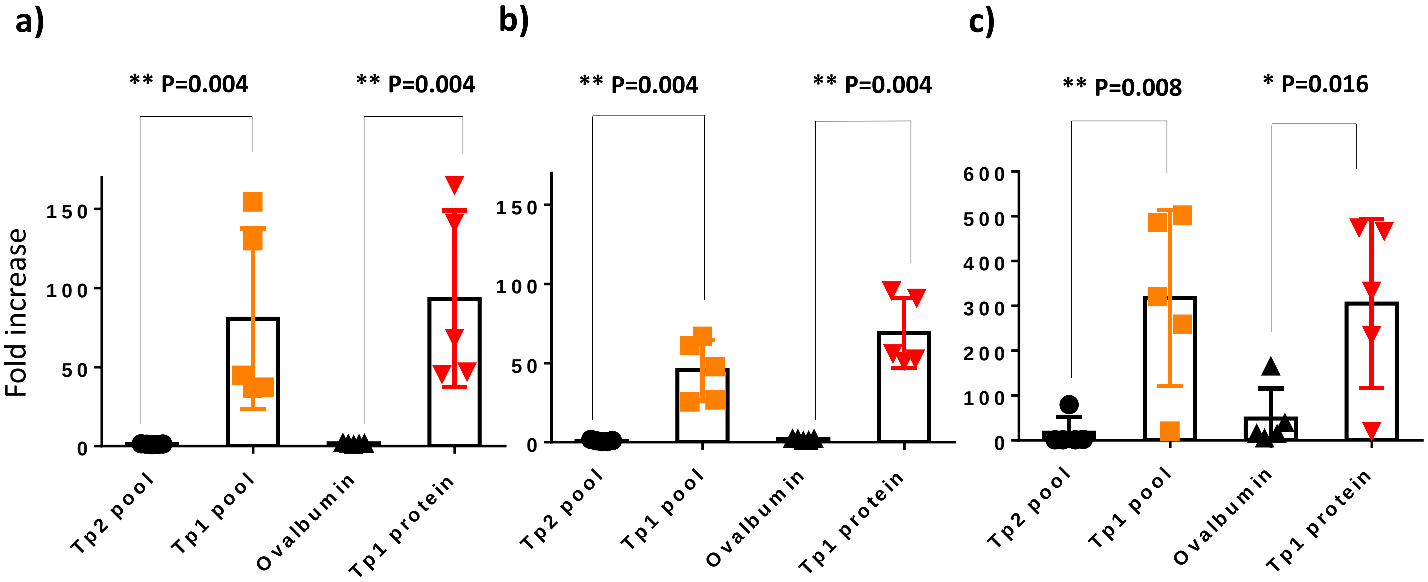

Supplementary figure 1. Tp1-specific proliferation of CD4$^+$ T lymphocytes induced by HAd5/MVA-Tp1 prime-boost immunization. Proliferation by ^{3}H-thymidine incorporation upon exposure to the Tp1 peptide pool and Tp1 recombinant full-length protein of purified CD4$^+$ T cells. **a)** group 1 (HAd-5/MVA-Tp1$_{tPA\text{-}SP}$), statistical analysis: Mann-Whitney, p = 0.004. **b)** group 2 (HAd5/MVA-Tp1$_{NO\text{-}SP}$), statistical analysis: Mann-Whitney, p = 0.004. **c)** group 3 (HAd5/MVA-Tp1$_{NAT\text{-}SP}$), statistical analysis: Mann-Whitney, p = 0.016. Proliferation is expressed as fold increase and compared to values obtained with irrelevant peptide pool or irrelevant protein and cells in media only.
